# Clinical Lecture

**Published:** 1858-06

**Authors:** 

**Affiliations:** Strasbourg


### CLINICAL LECTURE.

### *On Ready Diagnosis. By Professor Forget of Strasbourg.*

"Qui voit tout, abrege tout."

"The art of diagnosis consists essentially in the combination of all the elements which can aid, directly or indirectly, in the elucidation of diseases." This incontestible axiom is made to do good service by the investigators of our day, who are prone to exaggerate the value of each of the thousand minute details with which they are occupied, sometimes before their reality even has been sufficiently established. To this fever for inventions, this accumulation of hypothetical and often contradictory results, the name of "progress" is given. Be it so! Science indeed advances and is enriched daily; but it is in much danger of being buried beneath its mass of treasures, unless some one shall arrange them, assorting the crude products, and assigning to each its degree of value in every day practice. This nobody seems ready to undertake. Every one brings his grain of sand to the edifice, no one directs where it shall be placed; there are laborers in cohorts, but no architects, for we cannot so designate even the best of our authors of treatises on practical medicine. Once a book on practice was an event, and when Fernelious, Sydenham, Stoll, Borsieri, Cullen, or Ainel, published the results of long and conscientious study and meditation, their magistral work made a deep impress on the field of science, and commanded the attention of the world. Now-a-days ten neophytes set to work,* separately and silently, and relate what others do before they have learned what they will do themselves, commencing the novel by the last chapter, as the saying is, with the sole object of gaining by the speediest road a place at the professional banquet.

Hence we examine these compilations in vain for those large views which open up new horizons and strip the veil from obscurities. We find no harmonious whole, no lucid simplification. Open any modern pathological treatise you please at the article *Pleurisy*, for example, you will be told that inspection, palpation, mensuration, precussion and auscultation serve to diagnosticate this disease. The enumeration is exact, but as to any comparisons or reflections on the relative value of these various semeiotic means, they are not to be found, and the student is left to attach the same importance to dullness and to egophony; it will be unhappy for him, indeed, if the author does not insinuate that resonance and amphoric murmur are the rule, under

---

*It is scarcely necessary to remind the reader that a number of the recent German and French works on practice have been compiled by association of from three to twenty collaborators.—TRANS.

pretext that these phenomena have lately been observed in a few cases of pleuritus. Turn over to *Typhiod Fever:* you will learn that fever, prostration, delirium, the fuliginous tongue, cœcal gurgling, epistaxis, diarrhœa, etc., are the fundamental features of this affection, whence it results that all these symptoms have the same significance which is requisite in order to inculcate the idea of a general affection, *totius substantiœ.* When you encounter in practice typhoid fevers without a typhoid state, or frank phlemasiæ presenting the same phenominal portraiture as as is here ascribed to typhoid fever, you may get on as best you can. It is thus that confusion is eternalized; it is this want of comparison and logic which perpetuates chaos and maintains the authority of our modern augurs.

"A disease, that is to say, a group of morbid elements, being given, ascertain the order of their subordination over to the other," or the degree of influence which each of them exercises on the rest. The mode in which this problem has been comprehended and solved, is in truth the history of medical science, for it immediately conjures up every system of doctrine. According to the ideas which have prevailed on the nature and essential elements of disease, vitalism, humoralism, solidism, etc., have flourished or decayed. We do not propose to enter on this vast subject. We accept the notion of diseases commonly presented and ask for a thread to guide us through this immense labyrinth. In the first place, we may lay down: 1. That in a practical point of view there is no single essential element for all diseases, that is to say, that each of the exclusive doctrines is vitally defective and inadmissible; 2. That the principle elements vary or may vary in each class of diseases; thus in a given neurosis, the element strength or element debility may alternately denominate; 3. That this same variability, in a practical point of view, of the dominant elements, may present itself in each disease or in each particular patient. The demonstration of these propositions, which remove us far from the exclusive vitalist, organicist, and chemical doctrines, would embrace the entire field of pathology. We will attempt, however, to sketch a general outline to suggest the processes by which a solution of the proposed problem should be attained.

We know that there are dominant and subordinate, morbid elements. But our ignorance of the primary, essential, really dominant elements is often so great, that, to our astonishment, we often see a secondary or apparently accessory element assume a predominant importance in a therapeutic point of view. Such in a multitude of cases is the element *pain.* The toxic principal is the essential element in cases of poisoning and in contagious and infectious disorders, in syphilis, variola, typhus, etc. The rational indication is to eliminate or neutralize the toxic agent. But it is only in the minority of cases that we have the ability to do this; yet we still cure intoxications for which we have no direct antidote. Sometimes this is by allowing nature to act, as in the febrile exanthemata. In other cases, it is by combating the effects of poison, and keeping the patient alive till its virulence is exhausted, an inpoisoning by acrid or narcotic substances; and though, in these cases, we direct our attention to the secondary elements, to the inflammation or adynamia, the poison indirectly opposed is still the capital element. It is this that renders the maxim *naturam morborum curationes ostendunt* a most fallacious one, for it would require us to admit as many essential natures in the same disease as there are therapeutical methods applicable to it.

Having promised this much, let us enquire what are the sources whence the so-called primary or capital elements of diseases are to be derived. Of these sources, the most natural is unquestionably afforded by *etiology*, as is indicated by the aphorism *sublata causa tollitur effectus*, an aphorism as delusive as the last. We have shown elsewhere the obscurities and fallacies which often oppose the search for the real causes of disease, and have concluded that the causes of importance as regards treatment are constitutional causes difficult to reach, which happily do not always prevent recovery. So that this etiological doctrine, so imposing at the first glance, has a more limited application than is commonly imagined, as we have shown in speaking of poisoning. The most fruitful source whence important and positive elements of disease are to be derived is, despite what is said to the contrary, organic and functional *symptomatology*.

It is true that behind an inflammation or other organic lesion, there may exist, and often does exist, a general or special cause; but too often, alas! this cause is occult, and quite beyond the reach of remedies. We are forced then to come down to the obvious phenomena, and to accept them, at least provisionally, as primary elements, especially when the other phenomenal details of the disease naturally flow from them. Such are, in general, idiopathic inflammations; such are certain diathesic affections even, as tubercle and cancer; such certain functional diseases without appreciable material lesions, designated neuroses. The best that we can do in these cases is to attack the visible and tangible element, else we strike in the dark, and may deal a blow to the patient rather than the disease.

In a practical point of view, the so-called organic or symptomatic classifications of diseases are less viscious than they appear. They are as rational as circumstances permit, inasmuch as they express the positive facts, beyond which we can ascend only by inductions which practical experience and humanity condemn: *nil magni facias ex mera hypothesi.*

The *progress* of diseases is a more limited but less certain source of capital elements. Thus the intermittent character of disease is often of such predominant importance, as to be the one fact to be taken into consideration, as in pernicious fevers. The slow or rapid, the acute or chronic, progress of a disease is also a major element, which is the basis of most important practical demonstrations; for just as it is imperiously necessary to combat promptly and vigorously diseases which place life in immediate jeopardy, as pneumonia, peritonitis, or meningitis; so it would be most irrational to attempt to jugulate disease of slow progress.

The *terminations* of disease are likewise the source of important indications, according as they result fortunately by the unaided powers of nature as in erysipelas, simple icterus, etc., or lead to the grave complications, as in suppuration in pneumonia, aneurism in endocarditis, etc.

The ancients attributed great importance to *prognosis;* but prognosis is only a deduction from the causes, progress, and terminations, and consequently is comprised in these elements.

*Complications* constitute, in certain cases, elements of greater gravity than the primary disease itself: for example, the cerebral and pulmonary complications of typhoid fever, meningitis in erysipelas of the face, pericarditis endocarditis in articular rheumatism, etc.

Lastly, the *treatment* is the source of most important indications; for there is no better guide, practically, than the results of the medication employed, and nothing indicates more positively the necessity of a change of measures than the negative or injurious effects of those previously used. It is this incontestible principle which furnishes empiricism with the strongest argument it possesses.

From these brief considerations, it results that each element belonging to diseases may, in its turn, constitute the leading element. Hence the insufficiency of exclusive doctrines; for the relative importance of these varied elements cannot be predicated in advance and essentially depends on the peculiarities of each particular case.

Our present intention is to examine the relations of the elements belonging to the class of symptoms, which form the principal, though not unique basis, on which diagnosis is founded. The student who has been initiated into a knowledge of the characteristic signs of different diseases, by learning individual symptoms, should proceed to study the semeiotic elements, hitherto separately considered, in these relations to one another, in their rational connection, in a word. This intellectual labor constitutes what I call the ready method of diagnosis, which conducts us, by the shortest path, to a sufficent knowledge of the disease.

I use this term in opposition to the classical method, which arrives at a knowledge of disease only by reviewing methodically and minutely, in accordance with a given formula, all the details of the case. In instituting a parallel between these methods, I do not pretend that the former is surer than the latter, or sufficient for every case. On the contrary, the classic method is more complete and solid. But in a multitude of cases, an adequate knowledge of the case may be acquired, without pursuing the interminable meanderings of the classical process, and the latter can add nothing but a conjoinative evidence to the precise diagnosis already attained.

If the ready method had no other object than to abridge the examination, and exhibit the talent for observation and tact of the practitioner, its advantages would be small. But it spares the patient much fatigue, and relieves him from prolonged manipulations, dangerous mental and physical efforts and varied annoyances, entailed by the stereotyped examination in true classical style. Important facts are brought out clearly, stripped of superfluous accessors, and unobscured by erroneous or equivocal replies from the patient. Moreover, expeditious diagnosis is a practical necessity, not only in reference to the patient too feeble to comply with the exigencies of the classic mode, but in regard to the majority of practitioners, who either know, or, at all events, practice no other. It is a delicate subject to treat, but it must be admitted that the complete classic method is almost confined to the schools and the savans. It is notorious that, as soon as the students leave the benches, they forget the use and utility of the thousand minutiæ they have been taught. Pressed for the time, harassed by the difficulties, the prejudices, the vexations encountered at every step of practice, very many come to be satisfied with a ready method of diagnosis of almost any sort; and God knows what sad blunders result from this voluntary constrained negligence.

It is with a view of offering to practitioners of this class an equivalent, in facility, for their habitual methods, but an equivalent far superior in value and security, that I have undertaken these disquisitions on diagnosis; which I would term the elements of medical algebra, were not the metaphor too ambitious and false; false, inasmuch as our art can never pretend to the precision of mathematical science.

Without going back to Pinel, who seems to have first conceived the idea of framing a formula for the interrogation and examination of patients, who ever has turned over the pages of one of the innumerable clinical manuals, compends or treatises published in the last thirty years, must have noticed the formal systems of examination; which, under the pretext of preventing the practitioner from blundering, inveigle him into a labyrinth, and take him through endless windings to a gaol he might generally reach in a straight line. The laudable intentions of teachers have led, in fact, to those interminable books of questions, of which three-fourths, at least, are foreign to the case in point. I have never known a student or physician willing to load his memory with these ponderous protocols, even were they signed Bouilland or Piorry. For common sense teaches that the interrogation should be confined to subjects necessary for the elucidation of the case in hand, which is commonly to be attained by simple and natural means, as we shall see.

Thus, for what are termed the *preliminaries* of the case, the *sex* and *constitution*, we see the *age*, written on the features, need hardly be ascertained mathematically. *Menstruation*, *marriage*, *maternity*, the *number* and *health* of *children* and of *parents* are of importance in certain cases only. The *profession*, *regimen* and *antecedent diseases*, are common places of no value in a multitude of cases. None of these circumstances will lead us to the diagnosis of a disease, though many of them it is well to know, when the diagnosis is once established.

Coming now to the *present disease.* In many cases of external affections, medical or surgical, the disease stares us in the face, and abridges the interrogation greatly. Without speaking of traumatic lesions, all diseases affecting the skin and the mucous membrane accessible to the eye or touch: next all diseases which are reflected exteriorly: icterus, scurvy, chlorosis and other cachexies, typhoid diseases, convulsive and paralytic affections, diverse dropsies, etc., etc., all give their name at the first glance, and require but few questions. In some instances the sense of smell instructs us before or simultaneously with the sense of vision: as in gangrene, the puerperal state, cancer, the typhoid state, etc.

In the exclusively internal diseases, after a careful glance at the general physiognomy of the case, the first question should be: *Where do you feel pain?* the hand pointing out the exact seat indicated by the reply. The second question is this: *How long have you been sick?* and the reply informs you whether the disease is acute or chronic. Provided with these two notions, *seat* and *duration*, the examination already assumes a precise direction, and you proceed, without waiting for other information, to the exploration of the organ or apparatus supposed to be affected, adopting first those means which procure the most precise information, and often the diagnosis is evolved in this first enquiry.

Having ascertained the material or *organic* conditions of the disease, as far as possible, you proceed to investigate the

*functional* symptoms of the diseased organ, always in the order of their importance in a diagnostic point of view.

The disease once ascertained with precision, the hardest part of the task is accomplished. It remains to obtain information relative to the etiology, progress, complications, and therapeutic means, respecting all the anamnestics, in short that may be of utility. But the examination is concisely conducted in the directions indicated by the determined nature of the disease.

We see that, apart from the two questions already given, the examination is altogether a relative affair, and admits of no mixed formula. But, as we shall see, there are particular formulæ applicable to particular diseases, formulæ often susceptible of great abbreviation.

Having achieved the examination of the organ and disease, it is requisite to pass the other organs in review according to some settled plan, the choice of which is unimportant. Be careful to seek first for the capital symptoms, the presence or absence of which implies the integrity or alteration of the organ or function under examination. Thus the epiphenomena or complications are detected, and they must be looked for, even in the absence of suspicious symptoms.

But there are local diseases without pain, general diseases which cannot be accurately determined affections, in short, without local symptoms or seat; such are certain phlegmasiæ, termed latent, many organic lesions, some neuroses, intermittent fevers in the interval, certain humoral diatheses. We are then compelled to adopt the formal classic method, abbreviating as we can, and adhering strictly to enquiries relative to the organic and functional state, before descending to commonplace aimless questions. Example: a patient presents himself with no actual symptoms, and gives us no information. We proceed to the examination, and discover a tumefaction of the spleen. It is highly probable that the patient has intermittent, a fact determined by a few questions. Another subject presents no appreciable material lesion, and we can elicit no satisfactory replies; but a paroxysm soon supervenes of chill, and sweating, and again reveals to us the nature of the affection. Such cases are among the most unfavorable to the ready method; but in such the classical method does not shine. Another example; a woman reduced to marasmus has been attended by several physicians who have not ascertained the nature of her complaint. We examine the details of the case, and are not more fortunate than our predecessors. But the idea occurs to us to taste (!) the urine, and we detect a diabetes mellitus. In fact the sweet taste of the urine is a pathognomonic sign offering one of the most striking examples of ready diagnosis.

These enigmatical cases constitute what I call *veterinary medicine*, in which the physician, in absence of verbal information, is compelled to proceed as in the cases of animals. These cases are frequent; for they comprise, apart from stupid people, children, foreigners, delirious, apoplectic, asphyxiated patients, etc. etc. The best plan in every case is to proceed at once to search for the affected organs, by the material processes of palpation, percussion, ausculation, etc. These cases have not been sufficiently provided for by the classic method.

Diagnosis, in general, should be divided into positive or negative, according as its object is to determine the presence or absence of lesion. Positive diagnosis arrives at the most insignificant symptoms, and negative diagnosis elicits the organic or functional evidence which imply the integrity of organs. The latter often leads to the former, as when, without any preliminary indication of the seat of trouble, we are compelled to pass all the organic apparatus in review.

The most constant effect of an organic lesion is to produce functional derangement; therefore from the integrity of function, we infer the soundness of the organ. On this principle, the theory of negative diagnosis is founded on the determination of the most significant functions, just as positive diagnosis is based on the determination of the most expressive symptoms. This premised, I proceed to certain formulæ for negative diagnosis, applicable to the principal organs.

*Digestive apparatus.*—Have you appetite? How is your digestion: Are your bowels regular? affirmative replies on these three points afford a strong presumption that the disease is not located in the digestive organs; for there is scarcely any affection in the system, that does not modify the appetite, digestion, or defecation.

*Respiratory apparatus.*—Do you breathe well? have you a cough? Do you raise? Satisfactory replies make it probable that these organs are in a good state, for dyspnœa, cough and expectorations are inherent characteristics of most disorders of this apparatus.

*Circulatory apparatus.*—Do you feel your heart beat? This is about the only question you can address to common people; but is adequate in most cases, almost every heart disease producing palpitation or præcordial distress. The pulse and ausculation will clear up the diagnosis.

*Cerebro-spinal apparatus.*—Here questions are, strictly speaking, superfluous. The physiognomy, the speech, the gait and motion, almost always indicate at once whether nerve centres are intact.

*Apparatus of special Sensation.*—Do you see and hear well? Such are almost the only questions appertaining to this category; for the organs of taste and smell belong to the digestive and respiratory apparatus, and skin diseases reveal themselves.

*Urinary apparatus.*—Do you urinate without trouble? Is your water clear? These two questions are in general sufficient.

*Genital apparatus.*—Inspection, and the two preceding questions suffice in the case of males. In females, we enquire: Are you regular? Have you the whites? Replies to these questions convey what is essential respecting puberty, pregnancy the menopause, the different affections of the uterus and vagina; for disordered catamenia and vaginal discharges of some sort characterized almost all the sexual derangements of females.

We see by these examples how easy it is to arrive at a negative diagnosis, by keeping to the radical essential elements, which imply the rest. Of course, where practicable, the *ready diagnosis* should be corroborated by other questions and the usual methods of exploration. I wish simply to say that a negative diagnosis, sufficiently accurate for the generality of cases, may be attained very promptly. It should be an invariable rule to accompany or follow the interrogation by palpation and inspection of the organs examined. An abnormal swelling, in the provocation of pain by pressure may throw a flood of light on the morbid condition, which normal conformation and insensibility confirm the replies favorable to the idea of health.

*Virginia Medical Journal.*

---

**On the use of Iodide of Potassium in the Treatment of Leucorrhoea by Injections.**

